# Biotechnology and the transformation of vaccine innovation: The case of the hepatitis B vaccines 1968–2000

**DOI:** 10.1016/j.shpsc.2017.05.004

**Published:** 2017-08

**Authors:** Farah Huzair, Steve Sturdy

**Affiliations:** Science, Technology and Innovation Studies, University of Edinburgh, Old Surgeons' Hall, High School Yards, Edinburgh EH1 1LZ, Scotland, UK

## Abstract

The approval, from 1986, of a series of recombinant hepatitis B vaccines was a landmark both in the growth of biotechnology and in the development of the vaccine innovation system. In this paper, we show how the early development of the hepatitis B vaccines was shaped by a political and economic context that newly favoured commercialisation of academic research, including the appropriation and management of intellectual property; we elucidate the contingent interests and motivations that led new biotechnology companies and established pharmaceutical businesses to invest in developing recombinant vaccines specifically against hepatitis B; and we show how these and other factors combined to make those vaccines an unexpected commercial success. Broadening the scope of our analysis to include not just North America and Europe but also low- and middle-income countries, we show how the development of the hepatitis B vaccines facilitated the emergence of a two-tier innovation system structured by tensions between the demands for commercial profitability on the one hand, and the expectation of public health benefit for low- and middle-income countries on the other.

## Highlights

• The recombinant hepatitis B vaccines rehabilitated vaccines as commercially interesting pharmaceutical products.
• The recombinant hepatitis B vaccines helped substantially to establish the commercial viability of the biotech sector.
• The commercial success of the recombinant hepatitis B vaccines was largely unanticipated.
• The recombinant hepatitis vaccines helped establish a two-tier global vaccine innovation system.

## 1 Introduction

In May 1986 the vaccine Recombivax HB, which protects against hepatitis B infection, was approved for marketing in West Germany; approval by the United States Food and Drug Administration followed two months later. Recombivax HB marked a milestone in the development of medical biotechnology. Manufactured by Merck Sharp & Dohme, it was the first vaccine to be produced using recombinant DNA technology, and only the third recombinant product to be licensed for human use (following human insulin in 1982 and human growth hormone in 1985 and just preceding alpha interferon in June 1986). Two similar recombinant hepatitis B vaccines quickly followed: Engerix-B, marketed by SmithKline Biologicals, was approved in Belgium in December 1986 and in the US in September 1989; while GenHevac-B by Pasteur Vaccins was approved in France in May 1989.

Historians have paid little attention to the development of the hepatitis B vaccines – perhaps because the early biotechnology companies themselves tended to see them as scientifically and commercially less exciting than other first-wave recombinant medicines such as human insulin and interferon. As Nicholas Rasmussen notes in *Gene Jockeys*, these were high-profile medical molecules – insulin for its iconic position in the history of human physiology and medicine, and interferon because it was widely seen as a potential cure for cancer. As such, they were strong candidates with which to demonstrate the technical and commercial possibilities of the new recombinant biotechnology (Rasmussen, 2014). By contrast, the hepatitis B vaccines targeted a disease that at that time attracted little attention in North America and Europe. Accordingly, the vaccines ranked low among the priorities of the young biotech companies.

Seen in wider historical perspective, however, the recombinant hepatitis B vaccines turned out to be more consequential than the early biotechnologists and their business partners initially envisaged. Stuart Blume has argued that, between the 1960s and the 1990s, the “vaccine innovation system” underwent a major shift, from a predominantly publicly-funded, public-health-oriented enterprise in the years after the Second World War, to one dominated by private industry, including the new biotechnology sector, by the end of the century (Blume, 2008). Blume and Ingrid Geesink see the advent of the recombinant hepatitis B vaccines as symbolic of that shift (Blume & Geesink, 2000, pp. 50–51, 55–57). In the present paper, we show that it was not just symbolic; it was instrumental.

Drawing on a mixture of historical sources including published scientific literature, legal and policy documents, archives and oral history interviews, we construct an explanatory narrative of the development and commercialisation of the recombinant hepatitis B vaccines and the plasma vaccines that preceded them. We show how the early development of the vaccines was shaped by a political and economic context that strongly favoured commercialisation of academic research, including the appropriation and management of intellectual property (IP); we elucidate the contingent interests and motivations that led new biotechnology companies and established pharmaceutical businesses to invest in developing recombinant vaccines specifically against hepatitis B; and we show how these and other factors acted together to make those vaccines into an unexpected commercial success.

We go on to argue that this success served to embed vaccine development within a larger biopharmaceutical innovation system that increasingly involved collaborations between academia, new biotech start-ups and established pharmaceutical companies, mediated by strict control and licensing of IP. Finally, broadening the scope of our analysis to include not just North America and Europe but also low- and middle-income countries, we note how the development of the hepatitis B vaccines ultimately resulted in a distinctly two-tier innovation process, with the first tier, on which the present paper focuses, geared towards producing vaccines for sale at a substantial profit to rich-country markets, while a very different set of institutions developed to manufacture cheaper vaccines for use in poorer countries.

In telling this story, we contribute to the history of biotechnology as well as of the vaccine innovation system. As a number of analysts have emphasised, the growth of biotechnology involved significant reconfiguration of university-industry relationships, as university scientists and resources were increasingly engaged to supply the research expertise needed to realise commercial possibilities (e.g. Kenney 1986, Orsenigo, 1989, Vallas and Kleinman, 2008, Rasmussen, 2014). The story of the hepatitis B vaccines provides a case study of how that reconfiguration occurred around one particular set of products, highlighting the interaction of local contingencies with wider socio-technical factors which led to the institutionalisation of a particular field of biotechnological innovation. But focusing on vaccines also alerts us to the global dimensions of innovation, highlighting how, in this instance at least, the growth of biotechnology has tended to privilege the interests of wealthy countries and international businesses over the public health needs of low- and middle-income countries.

## 2 Hepatitis B and the plasma vaccine

In 1965 Baruch Blumberg, a geneticist researching variation in human disease susceptibility and immunity at the Institute for Cancer Research at Fox Chase, Philadelphia, published a paper detailing the identification of a previously unknown antigen in blood taken from an Aboriginal Australian (Blumberg, Harvey, Alter and Visnich 1965). Blumberg and others went on to show that this antigen was associated with hepatitis B infection, and subsequently that it was part of the virus itself – a small protein that eventually came to be known as the hepatitis B surface antigen (HBsAg) (Blumberg, 1977, Blumberg, 2003, pp. 72–118). This was cutting-edge research into a previously unknown pathogen, for which Blumberg was awarded the Nobel Prize in 1976.^1^ It also struck Blumberg as eminently applicable.

By definition, an antigen is a substance that provokes an immune response, and Blumberg quickly realised that HBsAg might be used to confer immunity against hepatitis B. In 1968, he and Irving Millman began developing a method of purifying HBsAg for use as a vaccine. Their approach was novel. Previous vaccines had been manufactured from killed or inactivated pathogens which, when introduced into the body, would provoke an immune response without causing the disease. Since the hepatitis B virus had proved practically impossible to cultivate outside the human body, it was not feasible to produce a vaccine in this way. Instead, Blumberg and Millman devised a method for purifying HBsAg from the plasma of hepatitis B carriers, while excluding or destroying any potentially infectious material by a complicated process of centrifugation and chemical treatment (Blumberg, 1977, p. 20). This was the first attempt to develop a vaccine involving only a subunit of the infectious agent. In October 1969 Blumberg and Millman filed a patent application with the US Patent Office – granted just over two years later – covering the vaccine and their production process (Blumberg & Millman, 1972).

These first steps toward creating a vaccine against hepatitis B owed much to policy push. By the late 1960s, the US National Institutes of Health (NIH), which funded Blumberg and Millman's work, was facing growing political pressure to demonstrate that its massive expenditure of public funds on research was delivering practical benefits (Berman, 2008, pp. 841–848; Yi, 2015, pp. 141–146). Millman directly attributed his and Blumberg's decision to develop and patent their vaccine to pressure from NIH (Millman, 2013, p. 140). Wider interest in developing a vaccine was muted, however. Hepatitis B was not a medical priority in North America or Europe at that time. Though recognized as a major public health problem in what was then called “the Third World”, in richer countries it was generally regarded as a “disease of outsiders” – sex workers, gay men and intravenous drug users – warranting little in the way of attention or resources (Muraskin, 1988, Stanton, 1994). Only in one setting did hepatitis B provoke particular alarm. During the late 1960s and early 1970s, a number of outbreaks of hepatitis B occurred in renal dialysis units, affecting not just patients but also practitioners. However, such outbreaks proved to be manageable by improved surveillance and infection control practices (Stanton, 1995, pp. 118–126).^2^ By comparison, medical practitioners and policy makers were inclined to regard vaccination as a high-cost, low-demand solution to a problem of marginal importance (Stanton, 1994, pp. 430–434).

At the same time, manufacturers were increasingly reluctant to invest in producing new vaccines. Since the 1950s, vaccine development had largely been sponsored by public-sector or charitable funders motivated by public health concerns. Such work was typically regarded as a public good, and was rarely subject to patent protection. Consequently, the pharmaceutical companies who were generally responsible for manufacturing and distributing vaccines made only limited profits from them. By the late 1960s, companies also faced additional disincentives to invest in vaccines. Increasingly stringent regulatory controls on the safety and efficacy of medicines, introduced by the US Food and Drug Administration (FDA) following the Harris-Kefauver Amendments of 1962, had added significantly to the cost of bringing new products to market; while companies were also increasingly aware of the risks of litigation associated with contaminated vaccines. By the late 1960s, many of the main pharmaceutical companies had withdrawn from vaccine development (US CongressOffice of Technology Assessment, 1979, Peretz, 1983, Blume, 2008, pp. 258–260). Consequently, when Blumberg and Millman began looking for a company to develop their vaccine and take it to market, they found themselves in a weak bargaining position. They were further hampered by constraints that NIH placed on the disposal of IP arising from the research it funded.

NIH's IP policy was informed by an expectation that the products of publicly-funded research should be developed in ways that best served the interests of the public. NIH drew two main implications from this. First, rather than allowing researchers or institutions to retain title over any patents arising from the research it funded, those patents should generally be assigned to NIH as custodian of the public interest. And secondly, when it came to licensing those patents, whenever possible they should be licensed only on a non-exclusive basis, since monopoly control of potentially life-saving products would empower manufacturers to exploit needy patients by charging excessively high prices (Berman, 2008, p. 843; Eisenberg, 1996, pp. 1671–1677; Metlay, 2006, pp. 568–581). This latter condition, in particular, was detested by the pharmaceutical industry, which had responded with what NIH's patents counsel would later describe as “a virtual boycott” of NIH-held patents (US Congress, 1976, p. 723), on the grounds that without the incentive of an exclusive license, it was not worth investing the funds necessary to bring a product to market.^3^

By the late 1960s, NIH faced growing political pressure to relax its IP policy. In 1968 the agency introduced a system of institutional patent agreements (IPAs) with universities whose patent policies NIH approved. These allowed universities not only to hold patents on NIH-funded research, but also to issue exclusive licenses where this was necessary to secure commercialisation – though NIH generally retained the right to veto such licenses if it thought that monopoly control of a particular invention was contrary to the public interest (Eisenberg, 1996, pp. 1682–1684; Mowery, Nelson, Sampat, & Ziedonis, 2004, pp. 87–88; Yi, 2015, pp. 145–146, 157). The Institute for Cancer Research was not among the institutions to be granted an IPA, however, and Blumberg and Millman therefore had to negotiate directly with NIH over the rights to the hepatitis B vaccine. NIH eventually awarded them foreign rights but retained possession of the domestic rights, which NIH insisted should only be licensed on a non-exclusive basis (Millman, 2013, pp. 141–142).^4^

Consequently, when in 1971 they began negotiations with Merck – one of the few US-based pharmaceutical companies that remained active in vaccine development, who were already considering hepatitis B as a possible target – Blumberg and Millman were unable to offer an exclusive license for the US market. Unsurprisingly, given pharmaceutical industry attitudes towards non-exclusive licensing, Merck turned them down. Negotiations with Merck would not reopen until early 1975, by which time the Institute for Cancer Research had obtained foreign patents in a number of European and Asian countries and was in discussion with Glaxo in the UK and the Institut Mérieux in France regarding oversees production. In August of that year, Merck finally agreed to a deal giving them all foreign rights to the vaccine as well as non-exclusive rights within the United States (Galambos, 1995, pp. 188–190; Millman, 2013, pp. 141–144; Muraskin, 1995, p. 7).

Once engaged, however, Merck moved quickly to commercialise the vaccine. The work was managed by Maurice Hilleman, widely regarded as one of the most accomplished industrial vaccinologists in the world. Hilleman's career in vaccine development dated back to 1944 when he had joined the virus laboratories of E.R. Squibb & Sons as a postdoctoral researcher. He joined Merck at the end of 1957 as director of a new department of virus and cell biology research, and by 1975 had secured the company's position as an international leader in vaccine development and manufacture (Offit, 2007). Hilleman had for some time been pursuing his own investigations into a possible hepatitis B vaccine (Hilleman et al., 1975), and quickly secured several additional patents for improvements on Blumberg and Millman's method of purifying HBsAg (e.g. Bertland et al., 1977, McAleer and Wasmuth, 1977), thereby consolidating Merck's control over the final product. Full-scale clinical trials began in 1978, and the vaccine was approved in the US in 1981 and launched under the trade name Heptavax-B in 1982 (Galambos, 1995, pp. 190–194).

The price of the new vaccine came as a shock to public health campaigners. Most vaccines sold at under $2 per immunisation – a price the World Health Organisation considered affordable for low- and middle-income countries (LMICs). By contrast, Heptavax-B was marketed in the US at around $90 to $100 for an immunising course of three doses, making it by far the most expensive vaccine produced until then, and placing it well beyond the reach of the vast majority of those most at risk from hepatitis B (Galambos, 1995, p. 194; Muraskin, 1995, p. 21). Hilleman defended the price on the grounds that the vaccine could not be produced for less. “Technologically, development of the vaccine was the most difficult challenge we have ever faced,” he observed, while the manufacturing process itself took fifty-six weeks for each batch from collection of carrier plasma to release of the purified, safety-tested product (Galambos, 1995, p. 193). But Merck's pricing policy was also consistent with a targeted approach to marketing, aimed at well-to-do consumers in wealthy countries – in particular healthcare workers and others at risk of occupational exposure to infected blood, but also gay men – who they anticipated could afford the vaccine either from their own pockets or through their insurance cover (Muraskin, 1988, pp. 287–288; Mamo & Epstein, 2014, p. 159; Stanton, 1994, pp. 435–439). In effect, Merck's efforts to secure and control intellectual property, and its decision to target wealthy consumers rather than LMICs, marked a shift away from the expectation that public health aims should take priority over commercial interests in vaccine innovation, and towards a concern with profit that was more in line with other kinds of pharmaceutical products.

In the event, take-up of Heptavax-B was low even among these target groups. Many feared that a plasma-derived product posed a risk of cross-infection with other pathogens – a fear which, although unsupported by evidence, gained salience with the appearance of HIV/AIDS and the scandal surrounding the infection of French haemophiliacs by contaminated blood products (Muraskin, 1988, p. 285; Conis, 2011, pp. 159–160; Stanton, 1994, pp. 439–440). Insurance companies too were unpersuaded of the benefits of hepatitis B vaccination, and refused to pay for Heptavax-B (Galambos, 1995, pp. 195–196). Merck's attempt to create a high-cost vaccine had foundered, confirming industry views about the unprofitability of the sector.

## 3 First steps towards the recombinant hepatitis B vaccines, 1977–1979

By the time Merck's plasma vaccine was approved for marketing in 1981, three different teams of scientists – located in Edinburgh, San Francisco and Paris respectively – were well on the way to developing alternative hepatitis B vaccines using recombinant DNA technology. Their work introduced a new production technology into the vaccine innovation system. But more than that, it also assimilated vaccines to a new biotechnology business model that was beginning to establish itself within the wider economy of pharmaceutical innovation. That business model had been made possible by the same shift in expectations regarding the commercialisation of publicly-funded research as had led Blumberg and Millman to patent their plasma vaccine. In particular, it was a product of the changing attitudes towards patenting that had driven the liberalisation of NIH's IP policy from the late 1960s, and that culminated in the passage of the Bayh-Dole Act of 1980 (Kevles, 1994, Berman, 2008, Yi, 2015, pp. 138–172).

Between 1972 and 1974, Herbert Boyer, a molecular biologist based at the University of California at San Francisco (UCSF), and his Stanford colleague Stanley Cohen had developed a powerful new technique for producing proteins using recombinant micro-organisms. Taking advantage of NIH's new, more liberal IP policy, Stanford and the University of California promptly applied for a patent. The application quickly became embroiled in wider debates about the propriety of claiming property rights in living organisms, and the first recombinant DNA patent would not be granted until 1980. Nonetheless, the universities' decision to seek a patent alerted Boyer, among others, to the commercial possibilities of the new technology, and the role that IP could play in exploiting those opportunities. In 1976, Boyer and venture capitalist Robert A. Swanson set up the biotechnology start-up company Genentech (Berman, 2008, Hughes, 2001a, Hughes, 2011). Their business model involved funding university-based researchers to develop and patent new recombinant methods of producing biopharmaceuticals. In the longer term, they hoped to build the company to the point where it would be able to manufacture the end products itself; but in the meantime, Boyer and Swanson planned to license their patents for development by larger pharmaceutical companies. In effect, Genentech turned patent-oriented biomedical research and the accumulation of biotechnological IP into a commercial value proposition in its own right (Doganova & Muniesa, 2015). That business model would be emulated by a number of biotechnology start-up companies established in Genentech's wake. It would also inform the development of the first recombinant hepatitis B vaccines.

### 3.1 Biogen

Created in 1978, Biogen was modelled directly on Genentech's approach to patenting university-based recombinant DNA research. Under the leadership of venture capitalists Ray Schaefer and Daniel Adams and the Harvard molecular biologist Walter Gilbert, Biogen looked beyond the US to recruit leading European molecular biologists to its scientific board. At a series of meetings in Geneva and Paris in early 1978, Biogen scientists identified a number of lines of recombinant DNA research they thought would lead to patentable products. Among them was a proposal by Peter Hans Hofschneider from the Max Planck Institute for Biochemistry in Martinsried near Munich, and Kenneth Murray from the Department of Molecular Biology at the University of Edinburgh, to develop a recombinant vaccine against hepatitis B (Hofschneider and Murray, 2001, Weissmann, 2001).

Advocates of the new recombinant DNA technology had from the early 1970s listed vaccines among the products that might be manufactured using recombinant methods. But Hofschneider and Murray also had personal reasons for pursuing a hepatitis B vaccine. Hofschneider had for some time been interested in the virus's role as a possible cause of liver cancer (Hofschneider and Murray, 2001, Neubert and Werner, 2004, p. 43). Murray, meanwhile, had direct experience of the disease itself. In 1969, two years after he arrived in Edinburgh, a particularly severe outbreak of hepatitis B had occurred in the dialysis unit at the city's Royal Infirmary (Stanton, 1995, p. 125). By the time the outbreak was contained in 1971, four members of staff and seven patients had died. Though Murray's department was not involved in the outbreak, he would remain poignantly aware of its effects on the local medical and scientific community.^5^

Hofschneider and Murray's initial research towards a vaccine proceeded quickly, with generous funding (by academic standards) from Biogen for facilities and staff (Hofschneider & Murray, 2001, pp. 46–47). Initially, Hofschneider had arranged to collaborate with a group of clinical researchers in Munich, who would supply serum from hepatitis-infected patients from which to source the virus. When that collaboration unexpectedly collapsed, Murray turned to colleagues in the Edinburgh University bacteriology department, which since the 1969 outbreak had become a major UK center of hepatitis B research (Hofschneider & Murray, 2001, p. 45). Increasingly, Murray now took charge of the vaccine work. Using state-of-the-art molecular biological techniques, he and his team not only cloned and expressed fragments of hepatitis B DNA in *E. coli*, but also painstakingly sequenced large sections of the viral DNA. In February 1979 they submitted a paper to *Nature* announcing that they had achieved expression of key parts of both HBsAg and a second antigen, the so-called core antigen, in *E. coli* (Burrell, Mackay, Greenaway, Hofschneider, & Murray, 1979). Six months later they submitted a second paper documenting the nucleotide sequence of 87% of the viral genome, including the DNA sequences that they believed to code for the core and surface antigens (Pasek et al., 1979). Meanwhile, in December 1978 Murray had filed a preliminary patent application with the UK Patent Office; and shortly after the second *Nature* paper appeared in December 1979, Biogen filed a full patent application with the European Patent Office, claiming broad rights over “Recombinant DNA molecules and hosts transformed with them which produce polypeptides displaying HBV antigenicity and genes coding therefor and methods of making and using these molecules, hosts, genes and polypeptides” (Murray & Schaller, 1987). Eventually granted in 1987, the patent gave Biogen ownership rights over viral DNA sequences that coded for the surface and core antigens, as well as over the manufacture of those antigens by recombinant methods.

### 3.2 Merck and UCSF

Meanwhile, a second project to develop a recombinant hepatitis B vaccine was under way on the west coast of the USA. Like the Biogen project, this was conducted in an academic environment with funding from a commercial company. In this case, however, the company was not a biotechnology start-up like Genentech, but the pharmaceutical giant Merck. The driving force within Merck was the physician and biochemist P. Roy Vagelos, who in 1976 had been recruited from an academic post to head the Merck Sharpe & Dohme Research Laboratory. Vagelos was keen to ensure that the company remain at the forefront of pharmaceutical science and technology, and in particular to explore the commercial possibilities of the new recombinant DNA techniques. With Hilleman's work on a plasma vaccine against hepatitis B already well under way within the company, and significant in-house expertise in handling both the virus and its constituent molecules, Vagelos identified the hepatitis surface antigen as a convenient target molecule with which to investigate the new production technology (Galambos, 1995, pp. 197–199).

Sometime in autumn 1977 Vagelos had a conversation with William Rutter – a molecular biologist and chair of the biochemistry department at UCSF in which Herbert Boyer worked. Like Boyer, Rutter was interested in exploiting the commercial potential of the new recombinant technology. He had reservations about Boyer's approach to commercialisation, however. As chair of the department, Rutter had devoted himself to building a distinctively collaborative, multi-disciplinary programme of research into molecular genetics (Jong, 2006).

While he supported Boyer's initiative to secure private funding for recombinant DNA research, he was concerned that Genentech did not adequately recompense UCSF for the benefit it gained from having access to university facilities and the wider programme of work under way there. Consequently, Rutter had declined an invitation to join Genentech, and instead tried to persuade the University to form its own technology transfer company to ensure that the profits of commercialisation were ploughed back into the institution itself (Hughes, 2011, pp. 80–82; Rutter, 1998, pp. 103–106, 175–176). Rutter's view of commercialisation thus had more in common with an earlier model of arm's-length, university-based non-profit organisations such as The Research Corporation and the Wisconsin Alumni Research Foundation, set up in the first half of the twentieth century to oversee commercialisation of publicly-funded research in ways that favoured the interests of university science over those of individual researchers (Apple, 1989, Mowery and Sampat, 2001).

In the absence of such an organisation at UCSF, in 1977 Rutter accepted funding from pharmaceutical company Eli Lilly to undertake research on recombinant insulin and growth hormone, in return for exclusive rights to any patents that might be generated (Rasmussen, 2014, pp. 62–63, 80, 83; Rutter, 1998, pp. 188–198). At the same time, aware of public concern about the possible risks associated with the new technology, Rutter had become “quite interested in developing a vaccine as a demonstration of benefit over risk” (Rutter, 1998, p. 159). His meeting with Vagelos in the autumn of 1977 revealed their common interest in hepatitis B, and led to an agreement between UCSF and Merck to develop a recombinant vaccine (Rutter, 1998, pp. 159–160, 188). As well as funding, Merck provided Rutter with hepatitis B DNA from Hilleman's laboratory, which Rutter passed to his colleague Pablo Valenzuela. By early 1979 Valenzuela had not only succeeded in cloning and expressing HBsAg in *E. coli*, but had also sequenced the section of the viral genome that coded for the antigen. The paper announcing these results appeared in *Nature* a week after Murray submitted his second paper to that journal (Valenzuela et al., 1979).

### 3.3 The Institut Pasteur

The third line of research into a recombinant hepatitis B vaccine took shape in the Institut Pasteur in France. The Institut differed, in organisation and orientation, both from the model of private-sector biotechnology represented by Biogen, and from the privately funded but academically based model of research and development represented by Rutter's programme at UCSF. A non-profit foundation, from its establishment in 1887 the Institut had combined commercial production of vaccines with high-quality microbiological and immunological research, often subsidised by substantial government funding (Liebenau & Robson, 1991). Work on a vaccine against hepatitis B fitted squarely into this programme.

Like Merck, the Institut Pasteur had a prior interest in developing a plasma vaccine against hepatitis B. From 1975, researchers at the Institut had been working with scientists and clinicians at the Faculty of Medicine in Tours to develop their own method of purifying HBsAg from the blood of hepatitis B carriers, using fractionation rather than the system of centrifugation and chemical treatment patented by Blumberg and Millman. In July 1980 the French researchers filed a US patent application for their new method (Maupas & Goudeau, 1980). By that time, clinical trials were under way, and the vaccine was licensed in France in 1981 under the proprietary name Hevac-B, manufactured and distributed by the Institut's own in-house company, Vaccins Pasteur (Chiron et al., 1998, Maugh, 1980, Maupas et al., 1976, Maupas et al., 1978).

As at Merck, researchers at the Institut Pasteur were conscious of the need to keep abreast of the latest in research and production technology. A key figure in this regard was Pierre Tiollais, a molecular biologist based at the Institut. Like Vagelos at Merck, Tiollais saw access to an in-house source of hepatitis B virus, and expertise in working with it, as invaluable resources to explore the productive possibilities of the new recombinant DNA technology. By mid-1979 he and his team had succeeded not only in cloning hepatitis B DNA in *E. coli* but also in sequencing the entire viral genome, as well as identifying those parts of the sequence that coded specifically for the surface antigen. The results were published in *Nature* just two months after Rutter and Valenzuela's paper appeared there (Galibert, Mandart, Fitoussi, Tiollais, & Charnay, 1979; also Charnay et al., 1979a, Charnay et al., 1979b).

## 4 Bringing the recombinant hepatitis B vaccines to market

Nicolas Rasmussen has argued that, for the biologists who formed the first generation of biotechnology companies, the opportunity to pursue interesting scientific research was as important as the as-yet-unproven possibility of a commercial pay-off (Rasmussen, 2014). This was clearly true of the early research into sequencing, cloning and expressing the hepatitis B surface antigen, which represented a substantial scientific achievement in its own right, resulting in four publications in *Nature* in the course of 1979 alone. But achieving expression of the surface antigen under laboratory conditions was one thing; achieving commercial production of an effective vaccine was another. Bringing the recombinant hepatitis B vaccines to market would require considerably more research and development, involving a good deal of trial and error, as well as skills in clinical research and marketing which went well beyond those of academic molecular biologists. Consequently, the subsequent development of the three recombinant hepatitis B vaccines depended heavily on the relationships the three groups of scientists were able to establish with commercial pharmaceutical manufacturers.

### 4.1 Merck and Chiron

The first major problem to confront all three teams was that the surface antigen produced by recombinant *E. coli* cultures did not stimulate a big enough immune response in experimental animals. Rutter and his team at UCSF were the first to identify a viable solution, though it took almost two years. Suspecting that recombinant bacteria produced the antigen in a different physical conformation from infected human cells, Rutter and his colleagues explored an alternative production method using a strain of human liver cancer cells that had been found to possess multiple copies of the hepatitis B genome. The UCSF scientists were able to culture these cells and induce them to secrete significant quantities of surface antigen, but subsequently dropped this line of development for fear that consumers would be suspicious of a vaccine produced from cancer cells (Galambos, 1995, pp. 196–197; Edman, Gray, Valenzuela, Rall, & Rutter, 1980). However, in 1981 Rutter and his team began collaborating with yeast geneticist Benjamin Hall of the University of Washington. They found not only that yeast could be induced to express the antigen in substantial quantities, but that the antigen was produced in a form that proved immunogenic when injected into rabbits. Their findings were announced at the International Congress of Virology in Strasbourg in August 1981 (Galambos, 1995, pp. 199–200; University of California at San Francisco, 1981, Valenzuela et al., 1982).

Shortly before making their findings public, Rutter and his team had filed an application for a US patent to cover the synthesis of hepatitis B surface antigen in recombinant yeast. The patents were to be assigned to the University of California (Rutter, Valenzuela, Hall, & Ammerer, 1988).^6^ By that time, however, Rutter was losing patience with the University and its failure to establish a technology transfer company to handle IP arising from research by its employees. With the commercial aspects of the hepatitis B work becoming increasingly onerous, and potential collaborators being offered positions in competitor companies, Rutter, Valenzuela and another collaborator, Edward Penhoet from Berkeley, formed their own biotechnology company, Chiron, in 1981 (Hughes, 2001b, pp. 100, 104–106; Hughes 2011, pp. 80–81). In keeping with Rutter's views on using private funding to support university research, they earmarked some of their stock “to be given back to the university, to recognize the general contribution the universities had made to the founding of Chiron. We … sort of recognized the parentage, if you will, of this whole thing” (Penhoet, in Hughes, 2001b, p. 108).

Meanwhile, with production of an effective recombinant vaccine now appearing increasingly likely, Merck moved on from simply funding Rutter's research at UCSF, and now began to bring the work in-house. In 1982 they recruited the molecular biologist and oncogene researcher Edward M. Scolnick to lead the company's programme in virus and cell biology, and specifically to develop new capacity in recombinant DNA technology. Following Vagelos's strategy of using the hepatitis B vaccine as a vehicle to explore the possibilities of the new technology, Scolnick focused first on the collaboration with Chiron, assuming personal oversight of the vaccine development work and bringing in additional scientists to help scale up production (Galambos, 1995, pp. 182, 200–203). Driven now by the resources of a major pharmaceutical company, production and clinical testing of the vaccine developed quickly. By mid-1984, the system of mass-producing surface antigen in yeast cultures was taking shape, and Merck were able to report that their first trials of the vaccine in humans had proved effective. Larger clinical trials quickly followed, and the vaccine – now branded as Recombivax – was approved for release, first in West Germany in May 1986, and then in the USA in July (McAleer et al., 1984, Schmeck, 1984, Jilg et al., 1984, Zajac et al., 1986, Galambos, 1995, pp. 203–204).

### 4.2 Biogen, Wellcome and SmithKline

In comparison to Merck's recombinant vaccine, commercialisation of the Biogen vaccine followed a rather more tortuous course. Like Rutter and the UCSF scientists, Murray and the Biogen team initially ran into problems with the limited immunogenicity of bacterially-produced surface antigen, and for a while they too looked into the expression of antigen in transformed mammalian liver cells, including human liver cancer cells (Gough and Murray, 1982, Koshy et al., 1983, MacKay et al., 1981). The UCSF team's announcement that a strongly immunogenic form of HBsAg could be expressed in yeast provided the way forward for Murray as for the Americans. Teaming up with Albert Hinnen, one of the scientists who had first demonstrated expression of recombinant DNA in yeast (Hinnen et al., 1978), Murray experimented with different DNA constructs to see which was most effective at expressing HBsAg. By early 1983 his team had developed a strain of yeast which produced acceptable levels of immunogenically-active surface antigen (Hofschneider & Murray, 2001, p. 52); and by August of that year, working now with experimental pharmacologist Huub Schellekens at the not-for-profit TNO Primate Center in the Netherlands, had demonstrated that the antigen prevented hepatitis B infection in chimpanzees (Hofschneider and Murray, 2001, Murray et al., 1984, p. 53). Announcing the findings, the British magazine *New Scientist* hailed “the first report of a successful test with the genetically engineered product in higher primates” (Anon, 1983).

The ability to produce a vaccine in the laboratory meant little if production could not be scaled up to a commercially viable level, however, and Biogen did not have the resources to take this forward on its own. Nor did the company see a hepatitis B vaccine as a priority in its portfolio of potential products. Despite the successful animal trials, the documentation produced for the company's initial public offering in 1983 made only passing mention of the vaccine, focusing instead on medicines such as insulin and interferon (Rasmussen, 2014). It was left to Murray himself to champion the vaccine project. In November 1983 he approached John Beale at Wellcome Biotechnology Ltd. in Kent, to propose a development deal. Beale was a leading expert in commercial vaccine development, who had led Glaxo's effort to commercialise the Salk polio vaccine during the 1960s. When Glaxo began to withdraw from vaccine research towards the end of the decade, Beale had moved to Wellcome Biotechnology (Day, 2006). Like Merck, Wellcome was one of the few large pharmaceutical companies that continued to pursue an interest in vaccines. Even so, Beale's initial response was cautious, noting that “We are attracted by this product opportunity but are keenly aware of the highly competitive nature of research and development in this area …”^7^ On the positive side, while the potential profits might be small, there were other strategic reasons for developing a hepatitis B vaccine, which the company “considered however to be the ‘entry ticket’ to newer markets e.g. the US”.^8^ Still, it was not until October 1984 that Wellcome and Biogen announced a formal agreement to develop the vaccine (Anon, 1984).

Once the deal had been struck, Beale moved quickly. Biogen had been conducting preliminary clinical studies as early as February 1984,^9^ and by June 1985 Beale was preparing registration batches of the vaccine and compiling the documentation necessary for full-scale clinical trials.^10^ The results of these trials were positive, and approval of the vaccine appeared imminent. But in the meantime circumstances at Wellcome had changed. By 1986, the Wellcome Trust, which owned Wellcome Biotechnology, was planning to sell off large parts of the company to raise funds for other activities, and was keen to maximise the value of shares in the company. In that context, the hepatitis B vaccine project looked more like a liability than an asset, and Wellcome withdrew from its agreement with Biogen.^11^

In the end, Biogen signed a new manufacturing agreement with SmithKline Biologicals, a Belgian subsidiary of the American company SmithKline Beckman, and one of the largest vaccine manufacturers in the world. The reasons for SmithKline's willingness to invest in the vaccine remain unclear, given the widespread doubts about its likely profitability – but the company may have decided to follow Merck and the Institut Pasteur in seeking to keep abreast of the latest production technology. In early December 1986 the Belgian authorities granted marketing approval for the SmithKline vaccine under the trade name Engerix-B (Ward, 1986). Approval of the vaccine for the US market would take longer: though a license application was submitted to the FDA late the following year, approval would not be granted until September 1989, by which time SmithKline was already marketing Engerix-B in more than sixty countries (Anon, 1988, Galambos, 1995, p. 204 n. 62).

### 4.3 Institut Pasteur

Third to market with a recombinant hepatitis B vaccine was the French team led by Tiollais. Following their sequencing of the viral genome, Tiollais and his team initially focused on developing methods of producing HBsAg in *E. coli*. By April 1981 their research had advanced sufficiently to file a US patent application specifying DNA sequences which coded for immunogenic fragments of HBsAg, and describing how the expression of those fragments could be improved by combining the viral DNA with certain vectors and promoter sequences (Charnay et al., 1984, Charnay et al., 1980). Tiollais too soon ran into the problem of the weak immunogenicity of bacterially-produced antigen, but unlike his competitors at Biogen and UCSF, he decided that mammalian cells rather than yeast offered a viable production system. Experimenting first with recombinant mouse cells, by 1982 he had shown that such cells not only produced HBsAg in significant quantities, but also that the antigen was effective in stimulating an immune response in rabbits (Dubois et al., 1980, Pourcel et al., 1982). A year later, Tiollais filed a second US patent application to cover this method of producing HBsAg; and two years after that he filed a third application detailing a particular combination of HBsAg with a human receptor molecule that further enhanced immunogenicity (Tiollais, Chany, Dubois, Pourcel, & Louise, 1994).

Over the next two years, Tiollais and his colleagues continued to experiment to determine which parts of the viral DNA should be included in the recombinant cells to produce the most effective vaccine (Milich et al., 1985), and what kinds of cells offered the most convenient and productive culture medium, settling eventually on Chinese hamster ovary cells. Not until April 1987 would the vaccine begin human trials (Anon, 1987). Manufactured by the Institut's own company Pasteur Vaccins under the trade name GenHevac-B, the vaccine was licensed in France in May 1989 (Girard, 1988, p. 758). For reasons that remain unclear, but may have to do with wider concerns about the safety of vaccines produced from mammalian cells, the company judged that GenHevac-B was unlikely to be approved outside France, so decided not to seek licenses elsewhere (Commission of the European Communities, 1994, p. 6).

## 5 The impact of the recombinant hepatitis B vaccines

Given industry perceptions that vaccines were generally unprofitable, and the limited uptake of Merck's plasma vaccine, initial expectations regarding the commercial performance of the recombinant hepatitis B vaccines were decidedly modest. As one of the Biogen scientists recalled: “[M]any people felt that the vaccines were not going to have the commercial value that some of the other [recombinant] products would do. They were somewhat de-prioritised.”^12^ This was reflected in the conservative way the new vaccines were marketed. Merck – the first company to bring its recombinant vaccine to market – simply pitched Recombivax as a replacement for its earlier plasma vaccine, targeting the same high-risk groups in rich countries and charging a “comparable” high price (Anon, 1986). SmithKline and Pasteur, as we shall see, took a broader view, including low- and middle-income countries in their marketing efforts. But their principal markets still lay in Europe and North America, where they followed closely on the pricing strategy set by Merck (Mowery & Mitchell, 1995, p. 984).

In the event, the new vaccines were far more commercially successful than anticipated. In part this was due to public perceptions of their safety. As early as 1979, in his original patent application for a recombinant method of producing hepatitis B antigens, Murray had declared that “the use of human sources for these antigens is disfavored because of the well recognized contamination problems in using human isolates” (Murray & Schaller, 1987, p. 4). In fact, experience would show that the plasma vaccine was generally very safe. Nonetheless, similar statements about the plasma vaccine were recycled in the press from 1983 onwards, as the recombinant vaccines entered first animal then human trials, then were introduced into practice. The very artificiality of the new vaccines now appeared as an advantage, offering a reassuring image of purity and safety (Conis, 2011, pp. 160–161).

At the same time, shifts in perceptions of the risks posed by hepatitis B infection helped drive the uptake of the vaccine. As evidence grew that the virus was a significant cause of liver cancer (Beasley, Lin, Hwang, & Chien, 1981), hepatitis B came increasingly to be seen, not just as a sexually-transmitted disease of outsiders, but as a significant public health risk affecting populations in wealthy as well as low- and middle-income countries. When, following a 1990 epidemic of measles, American public health officials launched a new programme of childhood vaccination to address a range of infectious diseases, hepatitis B was included in that programme (Mowery & Mitchell, 1995, pp. 987–988; Mamo & Epstein, 2014, pp. 159–160; Conis, 2011, pp. 162–164). With increasing numbers of American schoolchildren now expected to undergo hepatitis B vaccination, the market for the recombinant vaccines, and the profits they generated, far exceeded the manufacturers' early expectations.

The unexpected commercial success of the recombinant hepatitis B vaccines marked a turning point in the fortunes of the nascent biotechnology industry. From their creation in the mid-to late 1970s, the first wave of biotech start-ups had survived largely on the promise that they would eventually deliver profitable products. With the approval of Recombivax in 1986, however, Chiron became “one of the few biotech enterprises with a product to sell” (Fisher, 1986), and by 1991 was the first biotechnology company to turn an operating profit (Rasmussen, 2014, p. 122). In the case of Biogen, the success of Engerix-B helped to rescue the company from almost a decade of financial difficulties (Rasmussen, 2014, p. 181; Eccles, Nohria, & Berkley, 1992, pp. 104–106); according to one source, worldwide sales of Engerix-B approached $100 million in 1989 (Anon, 1990). For the big pharmaceutical companies, meanwhile, the success of the recombinant hepatitis B vaccines helped to rehabilitate the idea that vaccines could be worth investing in, paving the way for the development of other highly profitable vaccines, including the first “blockbuster” vaccines against human papilloma virus (Wailoo, Livingston, Epstein, & Aronowitz, 2010).

In boosting both biotech and big pharma interest in vaccines, the recombinant hepatitis B vaccines also effected a more general shift in the vaccine innovation system. Previously, vaccine research and development had been dominated by public and charitable funding, while the price of the resulting vaccines was determined in large part by the need to make them available to populations in low- and middle-income countries as well as the global North. By the 1970s, however, the diminishing profit margins associated with commercial manufacture and distribution of vaccines had led to all but a handful of companies withdrawing from the market. By contrast, the recombinant hepatitis B vaccines were predominantly a private-sector initiative, taking shape within and helping to articulate the developing network of interactions between academia, new biotech start-ups and established pharmaceutical companies that was coming to dominate pharmaceutical innovation more generally. They also displayed other features of the developing pharmaceutical innovation system – in particular the patterns of strict ownership and control of intellectual property that were crucial for mediating the increasingly complex network of institutional interactions that characterised it.

IP issues had been marginal to the earlier, predominantly public-sector vaccine innovation system. By contrast, as we have seen, all the teams involved in developing the recombinant hepatitis B vaccines applied for patents to protect their various inventions, leading to complex licensing arrangements when it came to manufacturing the vaccines. Merck and SmithKline, for instance, had to obtain licenses to three key recombinant hepatitis B patents assigned to the Pasteur Institute, the University of California and Biogen respectively, as well as patents on a range of other biotechnological and manufacturing processes (Mahoney, 2007, p. CS23) – a total of fourteen different patents in the case of SmithKline (Homma & Knouss, 1994, p. 52). To reduce transaction costs, Merck, Pasteur and SmithKline entered into a series of joint venture agreements covering not only cross-licensing of their respective patents, but also distribution of their vaccines in Europe and the USA. These agreements also had the effect of minimising competition between the three companies, while preventing other companies from entering the market (Commission of the European Communities, 1994). Consequently, despite the enormous growth in demand for the vaccine, the price fell only slowly: according to one report, by 1990 Merck was offering the vaccine to US public-sector providers at a price of around seven or eight dollars per shot, but continued to charge private-sector providers nearly double that amount (Asian Development Bank, 2001, p. 45). In effect, the recombinant hepatitis B vaccines conformed more closely to a pharmaceutical system increasingly characterised by the use of intellectual property to maximise profits than to an older, predominantly public-health-oriented system of vaccine development and distribution.

The high price of the recombinant hepatitis B vaccines also signalled a clear divergence between vaccine development for sale in wealthy countries and for low- and middle-income countries (LMICs). As Muraskin (1995) has shown, in many LMICs it was a plasma vaccine rather than one of the recombinant vaccines that first achieved wide distribution. As early as 1984, shocked at the price of the Blumberg-Merck vaccine, Alfred Prince, an expert in blood transfusion at the New York Blood Center, developed an alternative flash-heat method of purifying the surface antigen from plasma. Prince's method was simpler, faster and more resource-efficient than either Merck's or the Institut Pasteur's methods, producing larger quantities of vaccine from the same amount of plasma. Patenting his method but waiving royalties, Prince made it available to anyone who wished to produce a vaccine (Prince and Kwang, 1987, Muraskin, 1995, pp. 21–26, 60). In the absence of patent protection and faced with public anxiety about the safety of plasma vaccines, manufacturers in the USA and Europe were uninterested in marketing Prince's vaccine. In Asia, however, with support from an international task force of public health advocates, by the early 1990s a growing number of manufacturers were producing and distributing the plasma vaccine, prices had fallen below $1 per dose, and universal childhood immunisation against hepatitis B had become a realisable goal, at least for better-off Asian countries (Muraskin, 1995, passim; Maynard, Kane, & Hadler, 1989).

The new recombinant vaccines were much slower to enter that market. Both SmithKline and Pasteur certainly made efforts in that direction. Rather than reduce the price of their recombinant vaccines for sale in poorer countries, SmithKline, in particular, sought instead to represent the plasma vaccine as unsafe (Muraskin, 1995, pp. 202–210). Only with the emergence of local producers – particularly in India, where an intellectual property system that privileged process over product patents made it easier to circumvent European and American patent restrictions – did prices fall to more accessible levels; by 2001 at least ten producers, including three Indian companies, were supplying recombinant hepatitis B vaccines to domestic and export markets, at prices approaching those of the plasma vaccines (Asian Development Bank, 2001, p. 45; Chakma, Masum, Perampaladas, Heys, & Singer, 2011). In effect, the development and commercialisation of the hepatitis B vaccines was instrumental in the emergence of a distinctly two-tier vaccine innovation system: the first tier geared towards producing vaccines for sale at a substantial profit to rich-country markets; the second oriented toward producing cheaper vaccines for use in LMICs.

## 6 Conclusions

The development of the hepatitis B vaccines was instrumental in catalysing a major reconfiguration of the vaccine innovation system, first in the US and Europe and subsequently in LMICs. That transformation was largely unanticipated – an accidental consequence of local initiatives and innovations that only came together in the 1990s to create a coherent system. Seen in wider perspective, it was facilitated by a series of policy decisions that deliberately aimed to harness biological research to the commercial interests of the pharmaceutical industry. Our story is thus one of opportunistic innovation within a broader, enabling policy environment.

From the late 1960s, as we have seen, government-funded medical researchers came under mounting pressure to demonstrate that their work led to public benefit. At the same time, the very idea of public benefit was increasingly articulated in terms of the appropriation and commercial exploitation of intellectual property, especially by the pharmaceutical industry. The development of the Blumberg-Merck plasma vaccine represented an early, albeit ambivalent and ultimately unsuccessful attempt to realise this idea in the realm of vaccine innovation. The new vision of pharmaceutical commercialisation as a proxy for public benefit began to acquire greater solidity with the establishment of the biotechnology sector as an entrepreneurial intermediary between academic bioscience and the pharmaceutical industry. Bolstered by the Bayh-Dole Act and other relaxations in patent law, the biotechnology sector effectively incorporated intellectual property into a new asset-based business model for extracting commercial value from scientific research. In this setting, however, attention focused primarily on developing new biotechnological means of producing treatments such as insulin and interferon, which were expected to deliver substantial profits, rather than on vaccines, which were no longer regarded as profitable.

Consequently, efforts to develop recombinant vaccines against hepatitis B were initially motivated more by contingent local scientific and technical interests, including a desire among pharmaceutical manufacturers to keep abreast of the latest production technologies, than by any expectation of commercial success. In the event, the new vaccines confounded expectations by proving very profitable, alerting pharmaceutical manufacturers to the commercial possibilities that other such vaccines might offer. At the same time, the fact that a vaccine was among the first novel medical products to emerge from the new biotechnology sector did much to reinforce the perception among policy makers that commercialisation did indeed offer an effective means to deliver public health benefits, and that companies should be supported in this endeavour. Thus in 1994, the European Commission decided that while a joint venture between Pasteur Mérieux (successor to Pasteur Vaccins) and Merck to distribute the hepatitis B and other vaccines was anti-competitive and excluded other companies from the market, this was outweighed by the expectation that the Pasteur-Merck collaboration would accelerate innovation in an area of “genuine public health concern”, and so should be allowed to go ahead (Commission of the European Communities, 1994, p. 18). In effect, the successful commercialisation of the hepatitis B vaccines served to vindicate the expectations and interests that had informed policy on biomedicine and biotechnology since the late 1960s.

Finally, the development of the hepatitis B vaccines also throws light on the global relations of the emerging vaccine innovation system. Increasingly, this developed into a two-tier system. In North America and Europe, the latest biotechnologies were harnessed to produce new high-cost recombinant vaccines which served the demands of commercial profit as much as public health. Meanwhile, the public health needs of less wealthy countries were relegated to a second tier of institutions and initiatives which depended, at least initially, on cheaper plasma-based production technologies. Over time, a combination of philanthropic effort by non-profit organisations and the growth of local manufacturing capability resulted in a substantial transfer of recombinant hepatitis B vaccine technology from rich to poorer countries. Yet the relationship between the two tiers of the vaccine innovation system remains problematic.

As a number of critics have observed, international efforts to promote the roll-out of hepatitis B and other vaccination programmes, including expansion of indigenous vaccine production capacity, often proceed in the absence of demonstrated need or utility, and typically tend to favour the introduction of high-cost, high technology products over cheaper and more appropriate vaccines (e.g. Graham, 2016, Hardon and Blume, 2005, Madhavi, 2003, Madhavi, 2005). At the same time, IP regimes in LMICs are in many instances shifting to more closely resemble those in the global North, through enforcement of the Agreement on Trade-Related Aspects of Intellectual Property (TRIPS) of 1994, and as countries such as India seek to become global suppliers of pharmaceuticals in their own right (Greene, 2014, pp. 253–257; Sunder Rajan, 2011). As a result, not only is vaccine technology transfer becoming more difficult, but it is increasingly likely to favour the commercial interests of private corporations over public health concerns (e.g. Hendriks, 2012, Mahoney et al., 2004, Milstien and Kaddar, 2006). In effect, the commercial orientation that drove the emergence of a high-cost vaccine system in the USA and Europe is itself being transferred to LMICs at the expense of the kind of low-cost vaccines that might better serve the needs of local populations.

The development of the hepatitis B vaccines was pivotal in the assimilation of vaccine innovation to an emerging biopharmaceutical innovation system, including the creation of new global markets for recombinant pharmaceuticals. As such, it also exemplifies the tensions inherent in the new innovation system, particularly between the demands for commercial profitability on the one hand, and the expectation of public health benefit, especially in LMICs, on the other.

## Footnotes

1 The prize was awarded jointly to Blumberg and Carleton Gajdusek “for their discoveries concerning new mechanisms for the origin and dissemination of infectious diseases”. Gajdusek was included for his research into the prion disease kuru. “The Nobel Prize in Physiology or Medicine 1976”, Nobelprize.org, Nobel Media AB 2014, http://www.nobelprize.org/nobel_prizes/medicine/laureates/1976/, accessed 7 December 2015.

2 Two months before filing their vaccine patent, Blumberg and Millman had filed a US patent application for a test for hepatitis B (Coller, Millman, & Blumberg, 1975). This and other serological tests for HBsAg proved to be effective tools of infection control in healthcare settings, particularly in countries with strong public health networks (Blumberg, 2003, pp. 119–133; Stanton, 1995, pp. 126–137; Gerlich, 2013).

3 Industry resistance was driven especially by NIH's decision in 1962 to end the arrangement whereby companies screening products developed under NIH's Medicinal Chemistry Program were sometimes permitted to hold patents or exclusive licenses on chemicals they wished to develop commercially.

4 We have been unable to determine NIH's reason for withholding domestic rights. However, it may well have been because NIH regarded the vaccine as a public health measure, which should therefore remain in public hands, and should not be subject to a private monopoly.

5 Interviews with Sandra Bruce and John Pugh, 2015; lecture note “Hepatitis B – Looking at the Core of the Problem”: both in Kenneth Murray papers (Coll-1527), Edinburgh University Library Special Collections.

6 Rutter's claim to priority in achieving expression of HBsAg in yeast was contested by Ronald Hitzeman of Genentech. Hitzeman was working on expressing interferon, but had realised that a yeast expression system might also solve the immunogenicity problems confronting the vaccine researchers. The courts decided in Rutter's favour, on the grounds that he had been first to achieve rather than just conceive of producing a vaccine in this way (Hitzeman v. Rutter, 2001).

7 Letter from John Beale (Wellcome Biotech, Kent) to I.Buchanan (Biogen, Geneva), 7 November 1983, Kenneth Murray papers (Coll-1527), Edinburgh University Library Special Collections.

8 Internal communication to multiple Biogen members from Iain Buchanan (Biogen) reporting on a meeting with John Beale (Wellcome Biotech), 17 November 1983, Kenneth Murray papers (Coll-1527), Edinburgh University Library Special Collections.

9 Engerix-B, Summary for Basis of Approval, n.d., available online at http://www.fda.gov/downloads/BiologicsBloodVaccines/Vaccines/ApprovedProducts/ucm110155.pdf, accessed 28 December 2015.

10 Letter from Michael Winter (Wellcome Biotech) to Kenneth Murray, 12 June 1985, Kenneth Murray papers (Coll-1527), Edinburgh University Library Special Collections. An in-house volunteer study was underway at the Wellcome laboratories in Kent by September 1986: Progress report from M. Winter (Wellcome) to Biogen, 18 September 1986, Kenneth Murray papers (Coll-1527), Edinburgh University Library Special Collections.

11 Interview with Michael Winter, January 2015.

12 Interview with Michael Winter, January 2015.
